# Hemorrhagic fever viruses: Pathogenesis, therapeutics, and emerging and re-emerging potential

**DOI:** 10.3389/fmicb.2022.1040093

**Published:** 2022-10-25

**Authors:** Lizdany Flórez-Álvarez, Edmarcia Elisa de Souza, Viviane Fongaro Botosso, Danielle Bruna Leal de Oliveira, Paulo Lee Ho, Carlos Pelleschi Taborda, Giuseppe Palmisano, Margareth Lara Capurro, João Renato Rebello Pinho, Helena Lage Ferreira, Paola Minoprio, Eurico Arruda, Luís Carlos de Souza Ferreira, Carsten Wrenger, Edison Luiz Durigon

**Affiliations:** ^1^Institute of Biomedical Sciences, University of São Paulo, São Paulo, Brazil; ^2^Virology Laboratory, Butantan Institute, São Paulo, Brazil; ^3^Albert Einstein Institute for Teaching and Research (IIEP), Hospital Israelita Albert Einstein, São Paulo, Brazil; ^4^Hospital das Clínicas da Faculdade de Medicina, University of São Paulo, São Paulo, Brazil; ^5^Faculty of Animal Science and Food Engineering, University of São Paulo, São Paulo, Brazil; ^6^Scientific Platform Pasteur-USP, São Paulo, Brazil; ^7^Faculty of Medicine of Ribeirão Preto, University of São Paulo, São Paulo, Brazil

**Keywords:** hemorrhagic fever viruses, viral hemorrhagic fevers, fatal viral disease, emerging, re-emerging infectious diseases

## Abstract

Hemorrhagic fever viruses (HFVs) pose a threat to global public health owing to the emergence and re-emergence of highly fatal diseases. Viral hemorrhagic fevers (VHFs) caused by these viruses are mostly characterized by an acute febrile syndrome with coagulation abnormalities and generalized hemorrhage that may lead to life-threatening organ dysfunction. Currently, the events underlying the viral pathogenicity associated with multiple organ dysfunction syndrome still underexplored. In this minireview, we address the current knowledge of the mechanisms underlying VHFs pathogenesis and discuss the available development of preventive and therapeutic options to treat these infections. Furthermore, we discuss the potential of HFVs to cause worldwide emergencies along with factors that favor their spread beyond their original niches.

## Introduction

Hemorrhagic fever viruses (HFVs) are highly infectious RNA viruses that can lead to viral hemorrhagic fever (VHF) in humans. VHFs are mostly characterized by mild to acute febrile syndrome with coagulation abnormalities and generalized hemorrhage that can lead to multiorgan failure, and death (Schnittler and Feldmann, 2003; Basler, 2017). The frequency of hemorrhagic manifestations driven by HFVs can vary; however, they represent the acute form of the disease and one of the most common signs of the infection (Peters and Zaki, 2002; Paessler and Walker, 2013).

These viruses are a major concern to global public health because of their potential as bioweapons (Abir et al., 2022; Hickman et al., 2022) and the possibility to cause outbreaks with high fatality rates. In this minireview, we present the current knowledge regarding HFVs, including their common features and recently described pathogenic mechanisms underlying virulence leading to life-threatening infections. In addition, we present therapeutics and vaccines that are being used or under development to treat these diseases (Table 1). Finally, we highlight the potential of HFVs to transpose national boundaries and emerge as global threats.

**Table 1:** Common hemorrhagic fever viruses.

Family	Genus	Species	^a^Disease	Endemic regions	^b^Biosafety Class	Relevant viral proteins and roles on pathogenesis	Clinical signs and fatality rate	^c^Prevention	^d^Treatments	Ref
*Arenaviridae*	*Mammarenavirus* (New world)	*Argentinian mammarenavirus* (Junin virus)	Argentinian hemorrhagic fever (AHF)	South America - Argentina	3	NP (blocks IRF3 activation and translocation inhibiting IFN production and inhibits NF-κβ trough IKKε interaction); Z protein (inhibits NF-kappaB and IRF3 activation and RIG-I and MDA5 receptors and inhibits NF-κβ trough IKKε interaction)	Encephalitis, low viremia, hepatic deficiency; Fatality 15–30%	CANDID#1	Not reported	(Fan et al., 2010; Pythoud et al., 2012; Xing et al., 2015)		
		*Machupo mamarenavirus*	Bolivian hemorrhagic fever (BHF)	South America - Bolivia	4	Z protein (inhibits NF-kappaB and IRF3 activation and RIG-I and MDA5 receptors)	Petechiae, subconjunctival hemorrhage, hematuria, pulmonary edema; Fatality 30%	Not reported	Not reported	(Fan et al., 2010; Xing et al., 2015)		
		*Chapare mamarenavirus*	Chapare hemorrhagic fever (CHHF)	South America - Bolivia	4	Z protein (inhibits RIG-I and MDA5 receptors)	Skin hypersensitivity, diarrhea; Fatality 60%	Not reported	Not reported	(Fernandez-García et al., 2014; Xing et al., 2015; Plowright et al., 2019)		
		*Brazilian mammarenavirus* (Sabia virus complex)	Brazilian hemorrhagic fever (BzHF)	South America - Brazil	4	Z protein (inhibits RIG-I, NF-kappaB and IRF3 activation and MDA5 receptors)	Epigastric pain, bleeding gums, tonic–clonic seizures	Not reported	Not reported	(Fan et al., 2010; Xing et al., 2015)		
		*Guanarito mammarenavirus*	Venezuelan hemorrhagic fever (VHF)	South America - Venezuela	4	Z protein (inhibits RIG-I, NF-kappaB and IRF3 activation and MDA5 receptors)	Pulmonary congestion, renal cortical necrosis (autopsies); Fatality 33%	Not reported	Not reported	(Fan et al., 2010; Xing et al., 2015)	
	*Mammarenavirus* (Old world)	*Lassa mammarenavirus*	Lassa fever	West Africa -Sierra Leone, Liberia, Guinea and Nigeria	4	Z protein (inhibits RIG-I and MDA5 receptors)	Poor hemorrhagic manifestations, facial edema, pleural and pericardial effusion; Fatality 5–10%	rVSV∆G-LASV-GPC, MV-LASV	Ribavirin, Favipiravir, INO-4500, LHF-535	(Plowright et al., 2019)		
		*Lujo mammarenavirus*	Lujo hemorrhagic fever (LUHF)	South Africa- Zambia, Johannesburg	4	Z protein (inhibits RIG-I and MDA5 receptors)	Erythematous rash, no major hemorrhages; Fatality 80%	Not reported	Not reported	(Fan et al., 2010)		
		*Lymphocytic choriomeningitis mammarenavirus*	Lymphocytic choriomeningitis (LCM)	Southeast Europe	2	Z protein(inhibits RIG-I and MDA5 receptors impairing macrophages activation)	Meningitis, encephalitis, hydrocephalus; Fatality less than 1%	Not reported	Not reported	(Xing et al., 2015; Xing et al., 2015; Vilibic-Cavlek et al., 2021; Centers for Disease Control and Prevention C, 2022)
*Nairoviridae*	*Orthonairovirus*	*Crimean-Congo hemorrhagic fever orthonairovirus*	Crimean Congo hemorrhagic fever (CCHF)	Africa, the Balkans, Middle East and Asia	4	L protein (cleaves both Ubiquitin and Interferon stimulated gene 15 impairing IFN response)	Cutaneous ecchymosis, gastrointestinal and urinary tract bleeding; Fatality 30%	KIRIM-KONGO-VAX, MVA-based vaccine	Ribavirin, Methylprednisolone	(Scholte et al., 2017; Fillâtre et al., 2019; Temur et al., 2021)
*Hantaviridae*	*Orthohantavirus*	*Andes orthohantavirus*	Hantavirus pulmonary syndrome (HPS)	The Americas	3	Gn (inhibits IRF3 phosphorylation); N (inhibits STAT-1 phosphorylation resulting in abrogation Jak/STAT signaling pathway); GPC (inhibits Jak/STAT signalling pathway); NS (suppresses signalling *via* RIG-I/MDA5)	Pulmonary edema and cardiogenic shock; Fatality 60%	Andes virus DNA vaccine	Ribavirin, Methylprednisolone	(Levine et al., 2010; Matthys et al., 2014; Singh et al., 2022)		
		*Sin nombre orthohantavirus*	Hantavirus pulmonary syndrome (HPS)	The Americas	3	Glycoprotein precursor, GPC (inhibits Jak/STAT signalling pathway)	Pulmonary edema and cardiogenic shock; Fatality 60%	Not reported	Ribavirin, Methylprednisolone	(Levine et al., 2010; Matthys et al., 2014; Singh et al., 2022)		
		*Choclo orthohantavirus*	Hantavirus pulmonary syndrome (HPS)	The Americas	3	Not described	Pulmonary edema and cardiogenic shock; Fatality 60%	Not reported	Ribavirin, Methylprednisolone	(Singh et al., 2022)		
		*Necocli orthohantavirus*	Hantavirus pulmonary syndrome (HPS)	The Americas	3	Not described	Pulmonary edema and cardiogenic shock; Fatality 60%	Not reported	Ribavirin, Methylprednisolone	(Singh et al., 2022)		
		*Puumala orthohantavirus*	Hemorrhagic fever with renal syndrome (HFRS): Korean hemorrhagic fever, epidemic hemorrhagic fever, and nephropathia epidemica	Eastern Asia, Balkans, western and central Europe adn western Russia	3	NS (inhibits RIG-I activation);GPC ((inhibits RIG-I activation); N (inhibits activity of Granzyme B and caspase-3)	Bleeding diathesis, DIC, renal failure; Fatality 15%	HTNV/PUUV DNA	Ribavirin	(Raftery et al., 2018; Solà-Riera et al., 2019; Gallo et al., 2021;Singh et al., 2022; CDC, 2022b)		
		*Seoul orthohantavirus*	Hemorrhagic fever with renal syndrome (HFRS): Korean hemorrhagic fever, epidemic hemorrhagic fever, and nephropathia epidemica	Eastern Asia, Balkans, western and central Europe adn western Russia	3	Not described	Bleeding diathesis, DIC, renal failure; Fatality 15%	Not reported	Ribavirin	(Singh et al., 2022; CDC, 2022b)		
		*Hantaan orthohantavirus*	Hemorrhagic fever with renal syndrome (HFRS): Korean hemorrhagic fever, epidemic hemorrhagic fever, and nephropathia epidemica	Eastern Asia, Balkans, western and central Europe adn western Russia	3	N (inhibits activity of Granzyme B and caspase-3 and prevents T cell response inducing the expression of PD-L)	Bleeding diathesis, DIC, renal failure; Fatality 15%	HTNV/PUUV DNA	Ribavirin	(Raftery et al., 2018; Solà-Riera et al., 2019; Gallo et al., 2021; Singh et al., 2022; CDC, 2022b)		
		*Dobrava-Belgrade orthohantavirus*	Hemorrhagic fever with renal syndrome (HFRS): Korean hemorrhagic fever, epidemic hemorrhagic fever, and nephropathia epidemica	Eastern Asia, Balkans, western and central Europe adn western Russia	3	N (inhibits activity of Granzyme B and Caspase 3)	Bleeding diathesis, DIC, renal failure; Fatality 15%	Not reported	Ribavirin	(Solà-Riera et al., 2019; Singh et al., 2022; CDC, 2022b)
*Phenuiviridae*	*Phlebovirus*	*Rift Valley fever phlebovirus*	Rift Valley fever (RVF)	Sub-Saharan Africa and in the Arabian Peninsula	3	NSm (inhibits apoptosis of infected cells); NSs (inhibits transcription of IFN genes and eIF2α phosphorylation and downregulates the protein kinase PKR)	Macular rash, loss of central vision, seizures; Fatality less than 1%	RVF MP-12, TSI-GSD 200, ChAdOx1 RVF	Not reported	(Won et al., 2007; Ikegami et al., 2009; Ikegami and Makino, 2011; Nielsen et al., 2020)
*Filoviridae*	*Ebolavirus*	*Sudan ebolavirus*	Ebola virus disease (EVD)	Center and west of Africa	4	VP35 (inhibits RIG-I activation *via* PACT, inhibits activation of IRF3 and supresses dendritic cells maturation); VP24 (inhibits IFN production *via* STAT1)	Maculopapular rash, gastrointestinal symptoms; Fatality 50–90%	rVSVΔG-ZEBOV, VSVG-ZEBOV, rVSVΔG-ZEBOV-GP, V920 (rVSV-ZEBOV-GP), ChAd3-EBO Z, MVA-BN-Filo, Ad26.ZEBOV, MVA-BN-Filo, Ad26.ZEBOV/MVA-BN-Filo, MenACWY, cAd3-EBOZ vaccine, GamEvac-Lyo	Zmapp, Remdesivir, MAb114, REGN-EB3, Convalescent Plasma, GS-5734, Favipiravir, ansuvimab, Inmazeb, Azithromycin, Sunitinib and Erlotinib, Atorvastatin and Irbesartan	(Basler et al., 2003; Luthra et al., 2013; Yen et al., 2014; Malvy et al., 2019; He et al., 2022)		
		*Zaire ebolavirus*	Ebola virus disease (EVD)	Center and west of Africa	4	VP35 (inhibits RIG-I activation *via* PACT, inhibits activation of IRF3 and supresses dendritic cells maturation); VP24 (inhibits IFN production *via* STAT1)	Maculopapular rash, gastrointestinal symptoms; Fatality 50–90%	rVSVΔG-ZEBOV, VSVG-ZEBOV, rVSVΔG-ZEBOV-GP, V920 (rVSV-ZEBOV-GP), ChAd3-EBO Z, MVA-BN-Filo, Ad26.ZEBOV, MVA-BN-Filo, Ad26.ZEBOV/MVA-BN-Filo, MenACWY, cAd3-EBOZ vaccine, GamEvac-Lyo	Zmapp, Remdesivir, MAb114, REGN-EB3, Convalescent Plasma, GS-5734, Favipiravir, ansuvimab, Inmazeb, Azithromycin, Sunitinib and Erlotinib, Atorvastatin and Irbesartan	(Basler et al., 2003; Luthra et al., 2013; Yen et al., 2014; Malvy et al., 2019; He et al., 2022)		
		*Bundibugyo ebolavirus*	Ebola virus disease (EVD)	Center and west of Africa	4	VP35 (inhibits RIG-I activation *via* PACT, inhibits activation of IRF3 and supresses dendritic cells maturation); VP24 (inhibits IFN production *via* STAT1)	Maculopapular rash, gastrointestinal symptoms; Fatality 50–90%	rVSVΔG-ZEBOV, VSVG-ZEBOV, rVSVΔG-ZEBOV-GP, V920 (rVSV-ZEBOV-GP), ChAd3-EBO Z, MVA-BN-Filo, Ad26.ZEBOV, MVA-BN-Filo, Ad26.ZEBOV/MVA-BN-Filo, MenACWY, cAd3-EBOZ vaccine, GamEvac-Lyo	Zmapp, Remdesivir, MAb114, REGN-EB3, Convalescent Plasma, GS-5734, Favipiravir, ansuvimab, Inmazeb, Azithromycin, Sunitinib and Erlotinib, Atorvastatin and Irbesartan	(Basler et al., 2003; Luthra et al., 2013; Yen et al., 2014; Malvy et al., 2019; He et al., 2022)	
	*Marburgvirus*	*Marburg marburgvirus*	Marburg virus disease (MVD)	South of Africa	4	VP40 (inhibits IFN production *via* Jak1 pathway)	Abdominal pain, diarrhea, ecchymoses; Fatality 90%	cAd3-Marburg, cAd3-EBO-S, VRC-MARDNA023-00-VP, VRC-MARDNA025-00-VP, MVA Multi-Filo Ebola	AVI-7288, galidesivir	(Valmas and Basler, 2011; Brauburger et al., 2012)
*Flaviviridae*	*Flavivirus*	*Alkhurma hemorrhagic fever* (AFD)	Alkhurma hemorrhagic fever (AFD)	Saudi Arabia	4	E (mediates the entry of the virus and cause tissue tropism)	Elevated liver enzymes, elevated blood urea; Fatality 25%	Not reported	Not reported	(Al-Tawfiq and Memish, 2017; Shah et al., 2018)		
		*Kyasanur Forest disease virus* (KFD)	Kyasanur Forest disease	South Western India	4	E (mediates the entry of the virus and cause tissue tropism); non-structural protein, NS5 (antagonizes IFN response by inhibiting JAK–STAT signaling)	Gastrointestinal tract bleeding, lungs consolidation; Fatality 3%	Formalin inactivated tissue-culture vaccine	Not reported	(Holbrook, 2012; Shah et al., 2018)		
		*Omsk hemorrhagic fever virus* (OHFV)	Omsk hemorrhagic fever	Western Siberia	4	E (mediates the entry of the virus and cause tissue tropism)	Mucosa bleeding, petechial hemorrhages; Fatality 2.5%	Not reported	Not reported	(Holbrook, 2012; Shah et al., 2018; Wagner et al., 2022)		
		*Yellow fever virus*	Yellow fever	Subtropical areas of Africa and South America	3	E (mediates the entry of the virus and cause tissue tropism)	Rise of hepatic enzymes, jaundice; Fatality 30%	17D Yellow fever vaccine, 17D YF Vaccine plus Ig, Yellow Fever 17DD, HydroVax-002 YFV, vYF, Stamaril, SII-YFV, XRX-001 Inactivated yellow fever, MVA-BN-YF, Yellow fever vaccine (produced on serum-free Vero cells), YF-VAX	Galidesivir, TY014, Metformin Hydrochloride	(Monath and Barrett, 2003; Litvoc et al., 2018; CDC, 2022c)		
		*Dengue virus*	Dengue fever	Americas, Africa, the Middle East, Asia, and the Pacific Islands	2	NS2A (Inhibits RIG-I signaling by blocking TBK1/IRF3 phosphorylation); NS2B (Inhibits type I IFN production by cleaving human STING); NS5 (Inhibit IFN production *via* STAT2); NS4B (Inhibits RIG-I/MDA5 signaling by blocking TBK1)	Arthralgia, myalgia, rash; Fatality 20%	TV005, Tetravalent Dengue Vaccine (TDV), rDEN2Δ30–7,169, Dengue 1,2,3,4 (attenuated) vaccine, TetraVax-DV Vaccine TV003, 9vHPV, V181, YF-17D, IC14, T-DEN F17, T-DEN F-19, DTaP IPV//Hib vaccine, CYD, CYD 5555, CYD 5553, CYD 4444, TDENV-PIV	Ivermectin, JNJ-64281802, Montelukast, AT-752, Melatonin, Hypertonic sodium lactate, activated recombinant human factor VII, Chloroquine, Celgosivir, Modipafant, Ribavirin	(Mazzon et al., 2009; Aguirre et al., 2012; Dalrymple et al., 2015; Wilder-Smith et al., 2019; CDC, 2022d)

This table was constructed based on information at The International Committee on Taxonomy of Viruses (ICTV; The International Committee on Taxonomy of Viruses (ICTV), 2022) and includes the predominant hemorrhagic fever viruses. ^a^Data source: CDC.^b^Data source: Biosafety in Microbiological and Biomedical Laboratories (BMBL), 6th Edition (CDC, 2022e). ^c,d^Includes clinical trials of phases 1, 2 or 3 and approved vaccines or therapeutics. Clinical trials of phases 2 or 3 and approved vaccines or therapeutics are indicated for Ebola and Dengue fever virus diseases. Data bases: https://clinicaltrials.gov/ct2/home (NIH, 2022), https://www.clinicaltrialsregister.eu/ctr-search/search (EMA, 2022), https://trialsearch.who.int/ (WHO, 2022b).

## Clinical presentation and pathogenesis

**Figure 1:**
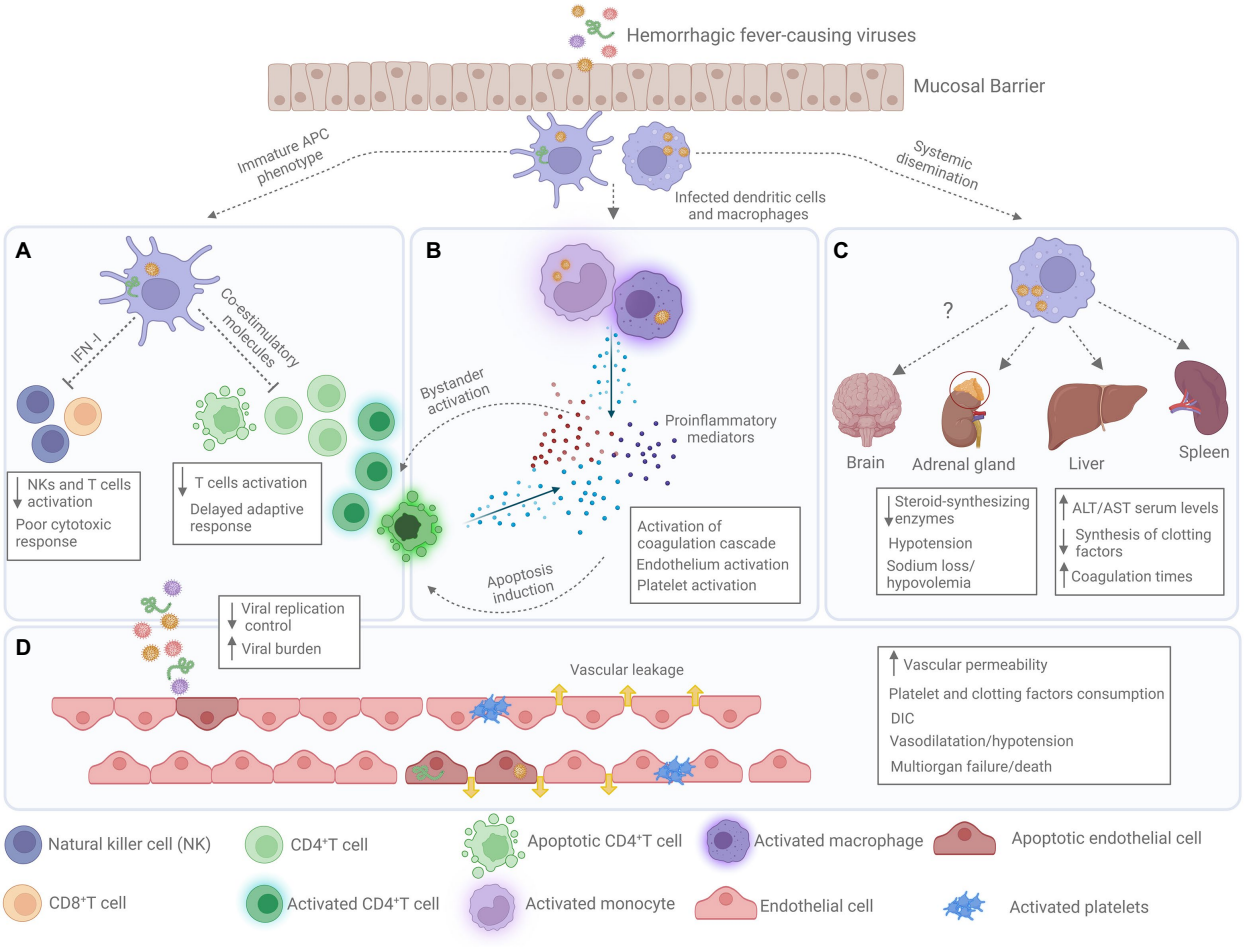
Pathogenesis of severe viral hemorrhagic fever. **(A)** Antigen presenting cells (APCs) present an immature phenotype with low expression of co-stimulatory molecules and low type I interferon (IFN-I) production, leading to poor NK and T cells activation causing a generalized immunosuppression, which lead to uncontrolled viral replication and high viral burdens. **(B)** During the infection monocytes and macrophages produced large quantities of proinflammatory mediators causing the phenomenon known as cytokine storm. These proinflammatory mediators cause endothelial and platelet activation and induce bystander activation and apoptosis of T cells. **(C)** Infected APC transport Hemorrhagic fever viruses (HFVs) to lymph nodes and other organs causing a systemic dissemination. Infection of some organs such as liver and adrenal gland results in high serum levels of hepatic enzymes (aspartate amino transferase-AST, and alanine amino transferase-ALT), low production of clotting factors and hypovolemia. **(D)** Sustained proinflammatory state and viral infection of endothelial cells induce abnormal vascular regulation and organ effusion, hemorrhages, and disseminated intravascular coagulation (DIC).

VHFs are characterized by abnormal vascular regulation and damage (Schnittler and Feldmann, 2003). Despite sharing some clinical manifestations, the cell and organ tropism, as well as the molecular mechanisms underlying their pathogenesis vary according to the causative agent (Peters and Zaki, 2002; Paessler and Walker, 2013; Table 1). However, all of them, target the cells responsible for initiating the antiviral response, causing a delay in the immune response. This delay, leads VHF patients to present high viremias and immunosuppression that can lead to a fulminant shock-like syndrome where inflammatory mediators play a major role (Marty et al., 2006; Reynard et al., 2014; Russier et al., 2014; Woolsey et al., 2022; Figure 1).

HFVs are transmitted through contact with or inhalation of contaminated materials from animal reservoirs or arthropod vectors; however, human-to-human spread through contact with infected blood and other body fluids is possible for most HFVs (Marty et al., 2006; Koehler et al., 2022). The incubation period varies from 2 to 35 days and begins with a prodromal period that typically last less than 1 week. This phase is followed by an increase in viral replication, which leads to an excessive release of cytokines, causing endothelial activation, increased vascular permeability, vasodilatation with subsequent hypotension, multiorgan failure, and death (Marty et al., 2006; Koehler et al., 2022).

Macrophages and dendritic cells (DCs) are the primary targets of most HFVs (Schmid et al., 2014; Huang et al., 2015; Schönrich and Raftery, 2019). Usually, despite being infected, these cells do not show an increase in activation markers or cytokine production. This immature profile of antigen-presenting cells (APCs) leads to deficient natural killer (NK) and T cell activation, impairing the induction of the subsequent immune response (Baize et al., 2004; Jin et al., 2010). Besides they can act as a viral reservoir and transport the virus into draining lymph nodes and other organs during immune patrol (Baize et al., 2004; Jin et al., 2010), explaining the high viral loads and systemic infection observed during VHFs (Reynard et al., 2014; Russier et al., 2014).

Several authors have reported that HFVs counteract the innate and adaptive immune responses in several ways. For example, Ebola virus inhibits DC maturation by the polyfunctional viral protein of 35 kDa (VP35; Jin et al., 2010), abrogating the adequate induction of adaptive responses. Other important players in the immune response, such as natural killer (NK) cells and gamma delta (γδ) T cells, decrease in number early during infection with pathogenic and non-pathogenic arenaviruses (Rodas et al., 2009), however, the underlying mechanisms are still unknown. Pathogenic arenaviruses are also able to suppress the type I interferon (IFN-I) response, avoiding interferon regulatory factor 3 (IRF3) activation and translocation to the nucleus through nucleoprotein (NP), they also inhibit RNA sensors activity by Z protein interactions (Pythoud et al., 2012; Rodrigo et al., 2012; West et al., 2014; Kang et al., 2021; Rojas et al., 2021). Inhibition of IFN-related signalling has been reported for other viruses as well; yellow fever virus (YFV) and dengue virus (DENV) inhibit the IFN response through the signal transducer and activator of transcription 2 (STAT2) and non-structural protein 5 (NS5) interaction (Fernandez-Garcia et al., 2016). This JAK/STAT pathway is also abroad by hantaviruses, which are also capable of inhibiting RIG-I-dependent IFN production, thus impairing the induction of adequate antiviral response (Levine et al., 2010; Gallo et al., 2021).

Not only the magnitude of IFN-I but also the T cell response have been associated with the disease outcome, where non-survivor patients may not generate an efficient immune response compared with the survivors, in whom the response is early and robust. Regarding T cells, both CD4+ and CD8+ T cells are important for protection against HFV. During infection, these cells show a predominant HLA-DR+ CD38+ phenotype, which is characteristic of an effector profile and differentiate towards a type I effector response characterized by IFN-γ production and cytotoxicity. Besides these effector mechanisms, CD4+ T cells are also involved in B-cell activation for antibody production (Farooq et al., 2016; Meyer and Ly, 2016; Ruibal et al., 2016; Speranza et al., 2018). However, during the infection the exacerbated response of these effector cells contributes to endothelial and hepatic damage, two hallmarks of the disease (Sung et al., 2012; Beier et al., 2015; Perdomo-Celis et al., 2019). On the other hand, regulatory CD4+ T cells, despite a conserved phenotype, showed a reduced number in peripheral blood, contributing to loss of activation control and leading to chronic inflammation (Zhu et al., 2009). This scenario leads to T-cell apoptosis, explaining the pronounced lymphopenia that characterizes most VHFs with the exception of those caused by hantaviruses, were lymphocytosis is normally observed (Marty et al., 2006; Rodrigues et al., 2012; Brisse and Ly, 2019). The lymphocyte apoptosis mechanism is not completely understood, but the involvement of TNF-related apoptosis ligand (TRAIL) and Fas/FasL (Fas Ligand) pathways have been proposed (Hensley, 2002). Aberrant responses with high expression of inhibitory receptors such as CTLA-4 and PD-1 have also been reported (Wauquier et al., 2010).

An increase in soluble proinflammatory mediators is another characteristic of VHFs. This proinflammatory state has been called “cytokine storm” and is characterized by the presence of high levels of IFNs, interleukin (IL)-6, IL-8, IL-10, IL-12, tumor necrosis factor-alfa (TNF-α), and reactive oxygen (ROS) and nitrogen (NO) species in serum (Messaoudi and Basler, 2015). As this phenomenon occurs despite the capacity of HFVs to suppress the immune response. It is unclear whether infected cells are the primary source of cytokines or are triggered *via* a bystander effect (Basler, 2017; Figure 1). However, some evidence suggests that infected monocytes and macrophages can be involved in the abnormal proinflammatory cytokine, chemokine, and ROS production in DENV and Ebola virus infections (Olejnik et al., 2017; Naranjo-Gómez et al., 2019). Direct interaction between the virus and T cells causing non-antigen specific activation and cytokine production has been reported for Ebola virus as well (Younan et al., 2017). Although these proinflammatory mediators are produced to control infection, at high and maintained levels, they exert cytotoxic effects by contributing to the death of bystander lymphocytes (Whiteside et al., 2018), tissue damage, loss of vascular integrity (Kang and Kishimoto, 2021), and hypotension mediated by NO (Li and Förstermann, 2000). Together, the information gathered to date suggests that an impaired and ineffective immune response leads to high viral loads and production of high levels of proinflammatory mediators, which are the main players in hemorrhages followed by shock with multiorgan failure that characterizes VHF clinical presentation.

Hepatic damage with high serum levels of alanine aminotransferase (ALT) and aspartate aminotransferase (AST), renal impairment evidenced by oliguria, coagulopathy, thrombocytopenia with prolonged coagulation times, and disseminated intravascular coagulation (DIC) also occur depending on the severity of VHFs (Figure 1; Mariappan et al., 2021). Some of these clinical signs can be explained as effects of “cytokine storm,” whereas others are related to direct viral infections facilitated by extravasation of infected monocytes/macrophages to tissues (Schnittler and Feldmann, 2003). During YFV infection, apoptosis and necrosis occur in up to 70% of hepatocytes and Kupffer cells, and viral nucleic acids and cellular infiltrates are observed in these tissues (Quaresma et al., 2007). Necrotic lesions have been found in the spleen, liver, bone marrow, heart, and kidneys during filovirus and arenavirus infection. Despite brain infection being observation only in animal models, survivors of Junín virus infection often develop neurological sequelae, suggesting an infection of the central nervous system (CNS; Paessler and Walker, 2013). Impaired endothelial function causes a wide spectrum of vascular effects observed during VHF, although the molecular mechanisms are largely unknown. According to experimental data, changes in the protein organization of tight junctions, particularly vascular endothelial cadherin (VE-cadherin)/catenin, are implicated (Mariko et al., 2019). Increased liberation of bradykinin, a potent inducer of vascular permeability, has also been reported in hantaviruses infections (Taylor et al., 2013). In addition, the virion glycoprotein (GP) of the Ebola virus has been shown to mediate abnormal activation and disruption of endothelial cells even in the absence of viral replication (Geisbert et al., 2003; Yang and Yang, 2021; Moni et al., 2022). Humans and nonhuman primates with VHF also present adrenocortical infection and necrosis, leading to impaired secretion of steroid-synthesizing enzymes that cause hypotension and sodium loss due to hypovolemia; these two important events have been reported in nearly all cases of VHF (Mariappan et al., 2021).

## Diagnosis and treatment

VHFs present with nonspecific symptoms making it difficult to clinically discriminate from other diseases, such as malaria, typhoid fever and even between the different causative agents of VHFs. This, together with the fact that VHFs also share laboratory parameters and the poor diagnostic value of serology given the impairment of APCs and lymphocyte functions during the acute phase of the disease (Feldmann and Geisbert, 2011); causes a delay in the diagnosis even after the fulminant disease process ensues (Racsa et al., 2016). Considering that, the viral genome detection is the better tool for diagnosis, however, sampling of blood requires medically trained personal and comprises important risks for the patient as well as for the health care personal (Racsa et al., 2016). Because of this situation traditional diagnosis has been restricted to large reference laboratories centered in Europe and the United States limiting the availability of the diagnosis in endemic areas (Racsa et al., 2016). Therefore, the sampling by non-invasive methods (e.g., saliva and/ or urine) might be a very valuable alternative. Currently, some laboratory diagnostic improvements are under development, including multiplex PCR, lateral flow assays and non-invasive sampling, including saliva and urine (Das et al., 2015; Niedrig et al., 2018; Barnes et al., 2020; Humaidi et al., 2021; Valinetz and Cangelosi, 2021).

For VHF treatment, two main components should be considered: (i) specific antiviral treatment and (ii) life support to prevent multiorgan failure (Ippolito et al., 2012). The appropriate treatment needs to be administered according to the phase of VHF: incubation, precoagulopathy, and coagulopathy (Ergonul et al., 2007). During incubation, post-exposure active/passive immunization and the administration of molecules with antiviral activity are the most effective approaches (Ippolito et al., 2012). Regarding active immunization, there are some approved vaccines against HFVs (Table 1). The YFV 17D, a live attenuated vaccine, has been administered around the world since 1932 with few side effects and providing life-long protection (de Menezes et al., 2015). The only vaccine successfully administered for an arenavirus is Candid #1, manufactured by the Argentinian government to prevent disease by the Junín virus (McLay et al., 2014). This vaccinal strain carries a mutation that attenuates the Junín virus as well as the Machupo virus infections, suggesting that it can provide cross-protection against arenaviruses (Patterson et al., 2014). There are no currently available Food and Drug Administration (FDA)-approved prophylaxis or vaccines for Crimean-Congo hemorrhagic fever (CCHF). However, an inactivated vaccine, developed in Bulgaria that induces strong humoral and T-cell responses has been used since 70s in the country (Mousavi-Jazi et al., 2012). There are other vaccines like Hantavax, that has been tested in patients with hemorrhagic fever and renal syndrome and has shown high seroconversion rates and a decrease in patient hospitalization (Cho et al., 2002; Song et al., 2016; Jung et al., 2018) and tetravalent DENV vaccine that has been shown to protect against severe forms of the disease for at least 5 years (Villar et al., 2015; Sridhar et al., 2018). Reassortant of Mopeia virus (MOPV) and Lassa fever virus (LASV) vaccine candidate, has been shown to protect against lethal LASV challenge in rodents. Viral particles produced by ML29-infected cells also interfere with the lymphocytic choriomeningitis virus (LCMV) carrier status, suggesting the potential of this formulation as a pan-arenaviral vaccine (Carnec et al., 2018; Johnson et al., 2019). Vesicular stomatitis virus (VSV) and adenoviral vectors have been evaluated in phase 1 studies and have been proposed as post-exposure vaccinations after accidental exposure of healthcare workers and laboratory workers to filoviruses (Tuffs, 2009; Ippolito et al., 2012; Ledgerwood et al., 2017; Regules et al., 2017). Recently, ERVEBO was licensed by the European Medicines Agency (EMA) and prequalified by the WHO as protective vaccination against Zaire ebolavirus. Additionally, in 2020, the EMA recommended granting marketing authorization to a second new vaccine called Zabdeno (Ad26.ZEBOV) and Mvabea (MVA-BN-Filo) delivered in two doses for individuals a year old and upwards (WHO, 2022a; Table 1).

Convalescent plasma therapy has been used as passive immunization and has proven to be effective in improving the clinical course and reducing mortality during hantaviral infection (Enria et al., 2008; Vial et al., 2015; Winkler and Koepsell, 2015). Additionally, Inmazeb, a mixture of three monoclonal antibodies against Zaire Ebola proteins received an orphan drug designation for the treatment of Ebola virus infection by the FDA (FDA, 2022). Finally, regarding molecules with antiviral activity. Nucleoside analogs have been tested against HFVs with promising results. Ribavirin has been effective against arenavirus and bunyavirus infection, reducing the mortality by 44% (Bossi et al., 2004; Soares-Weiser et al., 2010; Table 1), but other studies have shown insufficient efficacy of these compounds against hemorrhagic fever with renal syndrome (Malinin and Platonov, 2017). Favipiravir, one of the widely tested compounds in this group, has shown activity against several HFVs in animal models (Furuta et al., 2009; Safronetz et al., 2013; Madelain et al., 2017). These compounds can also be administered during the second phase of VHF, the precoagulophaty, when viral replication and high viremia occurs.

The third and last disease phase, the coagulophaty, is characterized by the onset of coagulation abnormalities driven mainly by the cytokine storm; immune-modulating drugs are the most effective pharmacological options during this phase. Factors that modulate coagulation, including recombinant inhibitors of factor VIIa and activated protein C, are associated with increased survival rates (Geisbert et al., 2003; Hensley et al., 2007). Inhibitors of platelet activation and macrophage migration have also been evaluated with promising results (Souza et al., 2009; Assunção-Miranda et al., 2010). Life support for patients with VHF and aggressive therapy to address multiorgan failure have proven to be lifesaving (Feldmann and Geisbert, 2011). Combined treatment with steroids, vasoactive drugs, hemodialysis, and mechanical ventilation resulted in favorable outcomes in patients infected with the Puumala virus battling multiorgan failure (Seitsonen et al., 2006). It has been reported that extracorporeal membrane oxygenation can also improve survival in patients with cardiopulmonary syndrome due to hantavirus infection (Dietl et al., 2008; Llah et al., 2018).

## Emergence and re-emergence potential

The ongoing recent trend of emerging and re-emerging diseases occurs almost every year. Influencing factors such as increased global population, aging, travel, and climate change, among other factors, favor the evolution and spread of new pathogens and the re-emergence of the older ones (Bloom et al., 2017). The distribution of HFVs, is closely linked to the ecology of their vectors and reservoirs such as rodents or arthropods. The changes in the distribution of these animal populations, recently well documented due to agricultural intensification and deforestation, represent a latent risk of re-emergence due to changes in the sites of circulation and spread of these viruses (Marty et al., 2006; Jones et al., 2013; Ellwanger et al., 2020).

For DENV and YFV, changes in the ecology of already established vectors and the appearance of other species with a better vectorial capacity can mark a greater expansion of these diseases and the occurrence of outbreaks in areas where they were already controlled (Brady and Hay, 2020; Pereira-dos-Santos et al., 2020). This was observed in Brazil, where major YFV outbreaks were observed in cities that were free of disease for almost 70 years due to viral adaptation to *Ae. Albopictus* (Amraoui et al., 2018). This vector has been identified as a potential player in emerging and re-emerging events because of its capacity to colonize natural breeding sites and ability to transmit more than a dozen arboviruses (Pereira-dos-Santos et al., 2020). The expansion of tick species into new geographic areas also represents a threat to the introduction of infections (Wikel, 2018). CCHF, the most widespread tick-borne viral disease affecting humans, has been restricted for many years to some regions of Africa, Asia, and eastern and southern Europe; however, its distribution is increasing rapidly in the eastern Mediterranean region due to the transport of tick vectors into these regions by birds (Al-Abri et al., 2017). The same phenomenon has been observed for other viruses, such as Alkhurma, a zoonosis associated with livestock in Saudi Arabia; it is now thought to be more widely disseminated than the initial focus in the Karnataka state in southwestern India because of abnormal vector migration (Mansfield et al., 2017).

In the case of rodents, their behavioral and demographic characteristics may contribute to their capacity to harbor, maintain, and spread the virus (Wikel, 2018). Rodents generally exhibit an r-selected life history, characterized by early sexual maturity and large litter sizes. This makes them vulnerable to resource depletion and climatic variation, impacting the prevalence of some RNA viruses in the human population (Easterbrook and Klein, 2008; Andreassen et al., 2021). The warming of the European climate has been predicted to increase the risk of hantavirus infection in the region and in the United States, where El Niño-related weather events have been linked to an increase in hantavirus pulmonary syndrome (HPS) incidence (Hjelle and Glass, 2000; Tersago et al., 2009). These data show how various external drivers create suitable conditions that allow zoonotic pathogens to expand and adapt to new niches. Often, these drivers are not just ecological but also economic, social, and political, functioning in various geographical and administrative territories (Wormser et al., 2010). Hence, the latent risk of re-emergence, the particular characteristics of this group of viruses, including their ability to cause disease with high mortality rates, the possibility of person-to-person transmission, and the easy dissemination through the air, have caused these viruses to be classified as bioweapon agents category A by the Center for disease Control and Prevention (CDC; CDC, 2022a).

This scenario, together with recent outbreaks affecting the lives and health of millions of people, highlights the need for improvement in global outbreak surveillance. A survey on laboratory preparedness for the response and diagnosis of CCHF conducted among the European Network for Diagnostics of ‘Imported’ Viral Diseases (ENIVD) members, revealed that despite more than 70% of laboratories having conditions to handle infectious samples, more than 60% emphasized the need for further training for laboratory workers, medical staff, and nursing staff (Fernandez-García et al., 2014). Detection of viral RNA combined with serology is performed routinely to ensure the early diagnosis of these diseases and is essential for effective surveillance, management of individual patients, and outbreak prevention. Nevertheless, progress in viral isolation, an essential part of virology studies is limited and consequently several aspects of the biology, ecology, and pathogenesis of HFVs remain unknown. In addition, treatment and vaccine development are rarely applied because high biocontainment laboratory (Biosafety level 4 - BSL4) facilities are deficient in endemic areas (Fernandez-García et al., 2014).

## Conclusion and future directions

Most HFVs cause high fatality rate due to their elevated pathogenicity, moreover, there are not many preventive vaccines and therapeutic options to treat VHF patients. This, together with the high risk of emergence and re-emergence of these pathogens, highlights the need of implementing several actions to suppress the latent risks that VHFs represent. These actions include the establishment of rapid and reliable protocols for laboratory diagnosis; guidelines for storage, processing, and transportation of samples; besides, conducting a comprehensive review of the BSL4 facilities suited to this work in the endemic areas, in addition to their capacities and capabilities, that will allow a safe management of these viruses (Fernandez-García et al., 2014). Is also necessary to study the spatial and temporal distribution of infection in reservoir populations, to predict interactions that favor spillovers (Plowright et al., 2019). These actions will allow to be prepared for future outbreaks reducing the impact of these diseases in human populations.

## Author contributions

ES, LF-A, CW, and ED conceived the manuscript. LF-A and ES wrote the manuscript. PH, JP, MC, VB, EA, HF, CT, GP, DO, LS, and PM contributed to manuscript revision and text editing. All authors contributed to the article and approved the submitted version.

## Funding

The authors would like to thank the Fundação de Amparo à Pesquisa do Estado de São Paulo (FAPESP) for financial support within the projects 2022/01812–8 (LF-A), 2015/26722–8, 2017/03966–4 (CW), 2020/12277–0 (ES), 2018/18257–1, 2018/15549–1, 2020/04923–0 (GP), 2020/06409–1 (ED), 2019/12303–4 (VB), 2017/27131–9 (PM), 2016/20045–7 (LS), 2016/08730–6 (CT); the Conselho Nacional de Ciência e Tecnologia (CNPq) for support within the projects 305,430/2019–0 (PH); 404,176/2019–4 (NIH project number 1 R01 AI149608-01; MC); 307,854/2018–3 (GP); 301,524/2019–0 (CW) and the Fundação Butantan for support (PH and VB).

## Conflict of interest

The authors declare that the research was conducted in the absence of any commercial or financial relationships that could be construed as a potential conflict of interest.

## Publisher’s note

All claims expressed in this article are solely those of the authors and do not necessarily represent those of their affiliated organizations, or those of the publisher, the editors and the reviewers. Any product that may be evaluated in this article, or claim that may be made by its manufacturer, is not guaranteed or endorsed by the publisher.

## Abbreviations

NP: Nucleoprotein
Z protein: Small RING finger protein
L protein: Large multifunctional protein
Gn: Viral envelope glycoprotein
N: Nucleocapsid
GPC: Glycoprotein precursor
NS: Non-structural protein
VP35: Viral protein of 35 kDa
VP24: Viral protein of 24 kDa
VP40: Viral protein of 40 kDa
E: Envelope protein
